# Cultural carcinogens in oral squamous cell carcinoma risk across global populations

**DOI:** 10.18632/oncoscience.662

**Published:** 2026-06-04

**Authors:** Nicole C. Nowak, Alyssa Forsyth, Brooke Blan, Shivani S. Ambardekar, Kelly Frasier, Nicole Werpachowski, Adrian P. Mansini

**Affiliations:** ^1^Rush Medical College, Chicago, IL 60612, USA; ^2^Texas College of Osteopathic Medicine, Fort Worth, TX 76107, USA; ^3^Midwestern University, Arizona College of Osteopathic Medicine, Glendale, AZ 85308, USA; ^4^Department of Medicine, University of Illinois at Chicago, Chicago, IL 60612, USA; ^5^Northwell Health, New Hyde Park, NY 11040, USA; ^6^Lenox Hill Hospital, Northwell Health, New York, NY 10075, USA; ^7^Department of Dermatology, Rush University Medical Center, Chicago, IL 60612, USA

**Keywords:** oral squamous cell carcinoma, cultural carcinogens, dermatology, prevention, global disparities

## Abstract

Oral squamous cell carcinoma (OSCC) is one of the most prevalent cancers worldwide, leading to significant illness and death, particularly in low- and middle-income countries. In addition to tobacco, alcohol, and human papillomavirus (HPV), several culturally ingrained practices, such as areca nut chewing, toombak, khat, reverse smoking, and hot mate drinking, remain underrecognized but important cultural factors contributing to OSCC globally.

These exposures operate through the formation of nitrosamine-induced DNA adducts, oxidative stress, chronic inflammation, and thermal injury. Areca nut and toombak are associated with oral submucous fibrosis and carcinoma, while reverse smoking and mate are linked to thermal and polycyclic aromatic hydrocarbon–related effects. Khat is connected to oxidative and cytogenetic damage.

This narrative review combines epidemiological, mechanistic, and public health evidence on cultural carcinogens in OSCC, emphasizing prevention strategies that honor cultural identity through education, harm reduction, and community involvement. Dermatologists specializing in mucocutaneous oncology can detect early lesions like leukoplakia, erythroplakia, and oral submucous fibrosis. Including oral mucosal screening in routine care provides an effective, affordable way to prevent diseases in high-risk populations.

Culturally sensitive, dermatology-focused prevention can help reduce OSCC incidence and disparities while respecting community identity.

## INTRODUCTION

Oral squamous cell carcinoma (OSCC) is a malignant tumor arising from the mucosal epithelial squamous cells that line the mouth and throat. It is the most common type of head and neck squamous cell carcinoma (HNSCC), accounting for at least 90% of cancers affecting the lips, buccal mucosa, gums, the front two-thirds of the tongue, the floor of the mouth, and the retromolar trigone [1, 2]. Globally, lip and oral cavity cancers caused 389,846 new cases and 188,438 deaths in 2022, ranking 16th and 15th in incidence and mortality, respectively [3]. There are significant disparities: Asia accounts for 66.3% of cases and 75.1% of deaths, whereas Europe accounts for 15.9% and 12.9%, respectively. All other continents contribute single-digit percentages. Notably, GLOBOCAN 2022 estimates that the annual number of cases will rise to 600,000 by 2045 [3].

In several regions, the elevated incidence of OSCC reflects the influence of culturally rooted exposure patterns that play a significant role in disease development. In South and Southeast Asia, where areca nut chewing is prevalent, oral cancer rates are among the highest globally, with India contributing a large proportion of global OSCC cases [4, 5]. In Sudan, the use of toombak, a highly carcinogenic form of smokeless tobacco, is strongly associated with oral squamous cell carcinoma. Epidemiologic studies demonstrate a 3- to 7-fold increased risk of OSCC and oral leukoplakia among toombak users compared with non-users [6, 7]. Similarly, khat chewing in parts of Ethiopia and Yemen has been linked to chronic mucosal irritation and premalignant oral lesions, although its direct carcinogenic role remains under investigation [8]. In Uruguay, Argentina, and southern Brazil, frequent consumption of very hot yerba mate has been linked to increased rates of oral and upper aerodigestive tract cancers, likely reflecting the combined effects of chronic thermal injury and exposure to polycyclic aromatic hydrocarbons [9]. Collectively, these culturally rooted practices illustrate how cultural behaviors shape global OSCC epidemiology and contribute to persistent geographic disparities in disease burden.

Carcinogen exposure significantly contributes to the development of OSCC. Besides tobacco, alcohol, and HPV, culturally ingrained practices such as areca nut chewing in South and Southeast Asia, toombak dipping in Sudan, and khat chewing in Ethiopia and Yemen are strongly associated with malignant transformation [10–13]. These practices cause DNA damage, chronic inflammation, and oxidative stress [14, 15]. Oxidative stress contributes to genomic instability, disrupts normal cell signaling, and promotes a tumor-supportive microenvironment [15]. These practices are also linked to pre-malignant fibrosis, further increasing malignant potential. Together, these findings highlight the need to consider both cultural and traditional risk factors in OSCC prevention [14].

Beyond oncologic and public health implications, these practices are highly relevant to dermatology. Dermatologists are often the first specialists to encounter oral mucosal lesions such as leukoplakia, erythroplakia, and oral submucous fibrosis during routine skin and mucosal examinations. Since many of these lesions appear at early, potentially reversible stages, dermatologists play a crucial role in their recognition, biopsy, referral, and culturally sensitive counseling. Dentists and oral health professionals likewise play a central role in the early detection of oral cancer through routine oral examinations, identification of suspicious lesions, and timely biopsy or referral, which can substantially improve outcomes when disease is identified at an early stage. Framing OSCC within the context of dermatology highlights the importance of awareness, training, and screening, particularly in diverse and immigrant populations where these practices are often underutilized.

## CULTURAL CARCINOGENS AND REGIONAL PRACTICES

### Areca nut chewing

Areca nut, derived from Areca catechu, has been used for centuries throughout South and Southeast Asia, with historical references dating back to the 1st century BCE [16]. Today, areca nut chewing, commonly in the form of betel quid, is practiced by an estimated 600 million people worldwide, often starting in adolescence [17]. The quid typically contains slaked lime and, in commercial products such as gutkha and pan masala, tobacco, which increase both addictive potential and carcinogenic risk [4].

Areca nut contains multiple alkaloids, polyphenols, and trace metals that contribute to the development of oral carcinogenesis. Among its alkaloids, arecoline is the most extensively studied and induces DNA strand breaks, micronucleus formation, and chromosomal aberrations. It downregulates tumor suppressors such as p53, stimulates reactive oxygen species (ROS), and activates oncogenic pathways including MEK, COX-2, and PI3K [4, 18]. During mastication, the nitrosation of areca and tobacco alkaloids generates nitrosamines, such as 3-methylnitrosaminopropionaldehyde (MNPN), N’-nitrosonornicotine (NNN), and 4-(methylnitrosamino)-1-(3-pyridyl)-1-butanone (NNK), the latter two of which are classified as Group 1 carcinogens by the IARC [19]. Slaked lime elevates oral pH, amplifying ROS production, inflammatory signaling, and nitrosamine formation. Areca nut itself contains trace metals, including copper, iron, zinc, and manganese, with copper present in relatively high concentrations compared to other plant-derived chewing substances. Elevated copper levels released during mastication have been implicated in the pathogenesis of oral submucous fibrosis (OSF), likely through stimulation of fibroblasts and increased lysyl oxidase activity, which promotes collagen cross-linking and extracellular matrix deposition [4]. Together, these mechanisms promote epithelial injury, fibrogenesis, and malignant transformation. Additional mechanistic details are summarized in Table 1.

**Table 1:** Mechanisms and epidemiologic evidence of cultural carcinogens in OSCC

Cultural practice	Main carcinogen/Exposure	Mechanistic pathways	Key epidemiologic evidence
Areca nut/Betel quid	Arecoline, nitrosamines (NNN, NNK), ROS, copper	DNA damage, p53 suppression, oxidative stress, fibroblast-to-myofibroblast transition (*TGF-β, α-SMA*), collagen cross-linking	Meta-analyses report increased OSCC risk among users (OR 2.8–7.5 vs. non-users)
Toombak (Sudan)	Extremely high TSNAs (NNN, NNK, NNAL)	DNA adduct formation, *RAS* and *TP53* mutations, microbial dysbiosis (Corynebacterium, Aspergillus)	Case–control studies report increased OSCC risk among toombak users (OR ~3–11 vs. non-users), particularly at quid contact sites; >30% of oral cancers in Sudan
Khat (Ethiopia, Yemen)	Mechanical trauma, ROS, cathinone metabolites	Oxidative stress, apoptosis, chromosomal instability (micronuclei), MAPK pathway dysregulation	Moderate evidence for premalignant oral lesions; association with OSCC in humans remains inconclusive
Reverse smoking (Philippines, Latin America)	Thermal injury, TSNAs, combustion products	Hyperthermia + carcinogen synergy, micronuclei formation, epithelial dysplasia	High prevalence of palatal lesions; OSCC prevalence reported up to ~18% in high-use communities
Yerba mate (South America)	Thermal injury (>65°C), polycyclic aromatic hydrocarbons (PAHs)	Oxidative stress, epithelial turnover, PAH-induced mutagenesis	Meta-analyses show increased oral/oropharyngeal cancer risk (pooled OR ≈2.1); strong dose-response with temperature and volume

Clinically, areca nut use is strongly linked to OSF and leukoplakia, both recognized as oral potentially malignant disorders (OPMDs). Recent proposals to reclassify OSF as areca nut-induced oral fibrosis highlight its distinct etiopathogenesis. Key mechanisms include oxidative stress, TGF-β–driven cytokine upregulation, and the fibroblast-to-myofibroblast transition, characterized by the expression of α-SMA and γ-SMA [18]. These changes lead to extracellular matrix buildup and progressive fibrosis, with malignant transformation rates ranging from 5.6% to 9.1%.

Epidemiological studies also show a rising rate of OSCC among younger groups in South and Southeast Asia, supporting the idea of early start and long-term exposure. Crucially, stopping areca nut use, whether or not it involves tobacco, lowers the risk of OPMDs and OSCC over time [20–22]. This timing emphasizes the importance of culturally tailored cessation efforts.

From a dermatologic perspective, areca nut-related lesions may appear as mucosal blanching, stiffness, or limited mouth opening, often identified during routine skin and mucosal checks. Dermatologists play a vital role in early detection, biopsy, and patient education, acting as key advocates for culturally sensitive prevention methods.

### Toombak

Toombak, a traditional smokeless tobacco product widely used in Sudan, is made from Nicotiana rustica, a species with an exceptionally high alkaloid content. Its fermentation process, alkaline pH (8–11), and nitrate-reducing microbial activity promote the formation of tobacco-specific nitrosamines (TSNAs), including NNN and NNK, at concentrations up to 20–560 times higher than Western smokeless tobacco products [22–24].

The method of use, which involves prolonged placement of a moist quid in the mandibular vestibule, results in sustained mucosal exposure to TSNAs and other carcinogens. These compounds undergo metabolic activation to form electrophilic intermediates, which then bind to DNA, triggering mutations in key regulatory genes such as RAS and TP53 [23]. Salivary TSNA concentrations in toombak users reach microgram-per-milliliter levels, among the highest reported for smokeless tobacco [25]. Clinically, these mechanisms explain the strong links between toombak and localized OSCC of the lip, buccal mucosa, and floor of mouth, with odds ratios as high as 11.0 among long-term users [6, 7].

*In vitro* studies support this mutagenic profile: toombak extracts cause DNA double-strand breaks, G2/M arrest, and apoptosis in normal oral keratinocytes, while dysplastic keratinocytes show some resistance but still exhibit persistent genotoxicity [26]. Microbial imbalance has also been linked, with increased levels of taxa such as Corynebacterium and Aspergillus in OSCC tissues from toombak users [27], indicating a possible synergy between microbial changes and chemical carcinogenesis. Table 1 summarizes the chemical, microbial, and genetic pathways involved.

For dermatologists, vigilance is essential in populations of Sudanese descent, where buccal mucosa leukoplakia or keratotic plaques may signal high-risk exposure, requiring biopsy and culturally sensitive cessation counseling.

### Khat chewing

Khat (Catha edulis) chewing is a cultural practice in East Africa and the Arabian Peninsula, especially in Ethiopia and Yemen, where fresh leaves are kept in the buccal vestibule for hours [28]. This causes chronic mucosal irritation, localized inflammation, and oxidative stress, creating a microenvironment that encourages epithelial dysregulation [29].

At the cellular level, khat extracts induce the production of reactive oxygen species (ROS), glutathione depletion, DNA damage, and apoptosis in oral keratinocytes and fibroblasts [29]. Micronucleus assays in habitual users reveal chromosomal instability, with increased aneuploidy and genotoxicity [30]. *In vitro* models demonstrate reduced epithelial thickness, premature differentiation, and dysregulated expression of cytokeratin 13, involucrin, and p21, which is mediated via the p38 MAPK pathway [31]. Elevated salivary malondialdehyde (MDA) and reduced antioxidant capacity among chewers further confirm oxidative imbalance [32].

Clinically, khat chewing is associated with keratotic mucosal lesions, most often on the chewing side, characterized by hyperkeratosis and atypia [33]. While mechanistic plausibility is strong, epidemiological evidence linking khat to OSCC remains limited and sometimes conflicting, with systematic reviews concluding moderate evidence for premalignant lesions but weaker support for direct carcinogenicity [34]. Table 1 outlines mechanistic and clinical evidence.

For dermatologists, especially in immigrant populations, awareness of khat use is important when evaluating persistent oral mucosal lesions. Early detection and culturally appropriate counseling may mitigate progression, particularly in settings where tobacco and alcohol co-exposure amplify risk.

### Reverse smoking

Reverse smoking, practiced in rural areas of India, the Philippines, and Latin America, involves placing the lit end of a cigarette inside the mouth. This exposes the palatal mucosa to intense heat and concentrated combustion products, producing a distinctive pattern of pathology [35].

Thermal injury induces epithelial thickening, keratosis, and atypia, while chemical carcinogens exacerbate DNA damage. Micronucleus assays demonstrate elevated genotoxicity in palatal mucosa, and salivary nitrosamine concentrations in reverse smokers reach several thousand parts per billion (ppb), far exceeding those of conventional smokers [36]. Clinically, over 90% of reverse smokers exhibit palatal lesions, ranging from fissuring and nodularity to leukoplakia and ulceration [35, 37]. Prevalence of palatal malignancy in some Colombian cohorts has exceeded 10%, among the highest reported globally [38].

Table 1 summarizes the thermal and chemical mechanisms driving these lesions. For dermatologists, recognizing palatal keratoses in older women from high-prevalence regions is crucial, as these lesions often represent the early stages of malignant transformation, requiring biopsy and intervention.

### Hot mate consumption

Hot mate, a traditional infusion brewed from Ilex paraguariensis, is widely consumed in Uruguay, Argentina, and southern Brazil, often multiple times daily at temperatures exceeding 65°C [39, 40]. The International Agency for Research on Cancer (IARC) classifies very hot mate as “probably carcinogenic to humans” (Group 2A) [40].

Carcinogenicity arises primarily from two sources: (1) thermal injury, which accelerates epithelial turnover, oxidative stress, and DNA damage, and (2) polycyclic aromatic hydrocarbons (PAHs) generated during smoke-drying of leaves, including benzo[a]pyrene [41, 42]. Comparative quantitative risk assessments, including margin-of-exposure (MOE) modeling that evaluates carcinogenic risk from PAH exposure versus thermal injury from very hot beverages, suggest that temperature-related injury is the dominant driver of carcinogenicity, although PAHs likely contribute additional risk [43]. Epidemiologic studies show dose–response relationships for volume, frequency, and duration of consumption, with synergistic risk when combined with tobacco and alcohol [9, 44, 45].

Table 1 outlines the key mechanistic and epidemiologic evidence. Clinically, heavy mate consumption is associated with elevated risk of OSCC and other upper aerodigestive tract malignancies. For dermatologists, awareness of this cultural practice is important when evaluating mucosal changes in South American and immigrant populations, where preventive counseling may include practical advice such as moderating infusion temperature.

### Cultural carcinogens: Summary

The cultural practices reviewed are deeply rooted in tradition, social rituals, and community identity. Despite their cultural significance, each of these exposures contributes to oral potentially malignant disorders (OPMDs) and OSCC. Importantly, these risks often manifest in the absence of conventional drivers such as cigarette smoking or heavy alcohol use [46].

The carcinogenic pathways are diverse yet convergent. Table 1 consolidates these mechanistic and epidemiologic findings, highlighting site-specific risks shaped by preparation, method, and duration of exposure, as well as synergistic effects with tobacco and alcohol.

While these practices fulfill important social and psychological functions, their persistence complicates efforts to prevent them. This duality underscores the challenge of balancing cultural preservation with cancer control. A nuanced approach is essential, one that respects cultural heritage while mitigating harm. The next section explores these obstacles in greater depth, examining the tensions between tradition and prevention, barriers to behavioral change, and the limitations of existing screening, education, and regulatory strategies in high-prevalence communities.

## CHALLENGES IN REDUCING CULTURALLY EMBEDDED CARCINOGEN USE

Efforts to reduce culturally embedded carcinogen use face multiple obstacles. These practices are deeply tied to cultural identity, social cohesion, and, in some cases, economic livelihood, making simple cessation campaigns both impractical and often culturally insensitive [47–49]. Public health messages that fail to account for these social dimensions risk resistance, mistrust, or outright rejection.

Studies investigating OSCC risk determinants outline geographical regional risk profiles by pattern, linked to both cultural practices and socioeconomic factors: variance in specific forms of carcinogen use or co-use, specific composition of carcinogen products, gender-specific carcinogen use norms, and socioeconomic status [50]. Museedi et al. explains OSCC risk related to regional standards of conduct, “The Middle East faces unique OSCC challenges related to specific culturally patterned risk behaviors. These practices-- defined as socially learned and transmitted behaviors that shape health and disease within a community…” [50]. Additional OSCC risk variables that highlight the need for further consideration and investigation of risk determinants outside of behavioral exposure include the highest level of educational attainment, location of residence, and dietary patterns [50].

Knowledge gaps further complicate prevention. Awareness of the carcinogenic risks of areca nut, toombak, khat, reverse smoking, and hot mate remains limited in many high-prevalence communities [51]. Even within healthcare systems, clinicians may under-recognize the significance of these exposures, leading to delays in diagnosis or inadequate counseling [52]. Resource-limited settings face additional barriers, including insufficient access to screening, limited biopsy capacity, and a lack of trained personnel [53, 54]. Additionally, although some carcinogenic pathways have been described for several of these practices, the mechanistic basis of others remains incompletely characterized. Future research should therefore prioritize larger epidemiologic and mechanistic studies to clarify causal pathways, quantify dose-response relationships, and better define their health impacts to inform prevention strategies in high-risk populations.

These obstacles are compounded by regulatory challenges. Commercial preparations such as gutkha and pan masala continue to be widely available despite bans in some regions, often through informal markets [55]. Similarly, enforcement of existing restrictions is inconsistent, undermining the effectiveness of policy measures.

From a dermatologic perspective, another challenge lies in clinical training. Oral mucosal screening is not consistently emphasized in dermatology practice, yet dermatologists are often the first to encounter lesions such as leukoplakia, erythroplakia, or oral submucous fibrosis [56]. Enhancing awareness and training in this area is essential to enable earlier recognition and culturally sensitive counseling [57].

Collectively, these challenges highlight the need for prevention strategies that extend beyond biomedical risk factors to incorporate cultural insight, socioeconomic realities, and dermatology’s unique role in early recognition. Table 2 summarizes barriers identified across regions and examples of attempted interventions.

**Table 2:** Barriers and culturally sensitive strategies for OSCC prevention

Intervention	Example/Context	Outcome	Limitations
Community-driven education	School and village programs in India, Sudan, Yemen	Increased awareness; reduced initiation among youth	Requires sustained local engagement; literacy barriers
Harm-reduction approaches	Lower temperature mate in South America; reduced frequency of areca nut use	Decreased mucosal injury and OPMD risk	Cultural attachment may limit adherence
Integration of screening	Oral mucosa inspection by dermatologists and primary care providers	Early detection of leukoplakia, OSF, erythroplakia	Limited training; competing priorities in busy clinics
Policy and regulation	Health warnings on areca/tobacco products; restrictions on sales to minors	Reduction in youth uptake; improved public recognition of risk	Enforcement varies; economic interests of producers
Community leadership involvement	Engagement of religious/community leaders to frame practices within health context	Greater acceptance of preventive messages	Success depends on trust and social cohesion

## STRATEGIES FOR CULTURALLY GROUNDED CANCER PREVENTION

Addressing culturally embedded carcinogens requires strategies that align health promotion with cultural identity. Interventions are most effective when they respect tradition, adapt to community values, and provide practical alternatives rather than relying solely on prohibition.

Community-based education has shown promise. In India, framing areca nut as a pediatric health risk helped reduce initiation among school-aged children [58]. In Guam and Saipan, the Betel Nut Intervention Trial (BENIT), a randomized community-based study evaluating behavioral interventions to reduce areca nut (betel quid) use among habitual chewers, found that culturally tailored cessation materials were significantly more effective than generic messaging in promoting quit attempts. Quit attempts occurred in 38.6% of participants in the intervention group compared with 9.1% in the control group (*p* = 0.0058) [59]. Similar approaches, linking health communication to local narratives, language, and cultural symbolism, consistently outperform standardized campaigns [60]. Harm reduction strategies may also be more acceptable than elimination. Examples include moderating mate infusion temperature, promoting smoke-free processing methods, or encouraging less carcinogenic substitutes for high-risk quid formulations [42, 61, 62]. Additionally, culturally sensitive prevention strategies include the endorsement of practices that promote oral health. Evidence suggests mouth rinsing immediately after product use, Mediterranean diet, dietary patterns favoring minimal consumption of processed foods or lower overall dietary inflammatory index scores, living in an urban residence, attaining a higher level of education, and Miswak oral hygiene practices may be protective against OSCC [50]. These practical approaches reduce risk while respecting cultural practices.

Policy interventions remain crucial, but they require community support. Partial bans on gutkha and pan masala, for example, have had limited success due to the presence of informal markets, highlighting the importance of consistent enforcement combined with culturally relevant education [55, 63]. Museedi et al. proposes policy change to combat smokeless tobacco consumption through its integration into the World Health Organization (WHO) Framework Convention of Tobacco Control (FCTC), favoring increases in product taxation, banning of all tobacco advertising, promotion, and sponsorship, and mandates regulating all forms of smokeless tobacco use in social settings, in addition to clearly visible product health warnings while offering support for product cessation [50, 64]. Table 2 summarizes examples of interventions across regions and their results.

Dermatologists can strengthen prevention by incorporating oral mucosal screenings into routine practice and offering culturally sensitive counseling [57]. Collaborating with community leaders, schools, and public health initiatives positions dermatologists not just as clinicians but also as advocates who can connect biomedical knowledge with cultural awareness. This dual role improves early detection and helps communities lower OSCC risks without dismissing their traditions.

## REFRAMING PUBLIC HEALTH: RESPECTING CULTURE WHILE REDUCING HARM

Dermatologists are uniquely positioned to bridge the gap between clinical detection and community prevention. As specialists experienced in examining mucocutaneous surfaces, they can identify early oral lesions such as leukoplakia, erythroplakia, and oral submucous fibrosis during routine skin or mucosal evaluations [57]. By incorporating systematic oral inspections, prompt biopsies or referrals, and culturally sensitive counseling into their practice, dermatologists contribute to early detection and preventive oncology. Integrating oral cancer awareness into dermatology and global health frameworks helps reduce the incidence and mortality of OSCC while respecting cultural identities [65] (Table 2).

Public health interventions must balance cultural respect with disease prevention. Practices such as betel nut chewing, khat use, or mate drinking are deeply interwoven with community life. Rather than pursuing eradication, effective strategies aim to preserve cultural meaning while minimizing harm. Successful models from Nigeria and Taiwan demonstrate that community-led engagement, facilitated through local leaders, workplaces, and religious networks, promotes long-term behavioral change and acceptance [66, 67]. Additionally, understanding socioeconomic risk determinants is critical to improving OSCC outcomes. In fact, studies have demonstrated an inverse relationship between socioeconomic status and oral cancer general awareness and knowledge about OSCC risk factors, signs, and symptoms [68]. Socioeconomic factors that have been linked to an increased risk of OSCC development include low education level, limited access to healthcare, and gender disparities corresponding to regional cultural norms [50]. Museedi et al. proposes tailored public health and clinical response recommendations, including identification of at-risk populations through targeted screening methods with practical intervention, such as mobile dermatology and/or dental units, in place [50].

By working with cultural stakeholders and creating region-specific educational materials, dermatologists can reshape prevention as a form of empowerment [20]. This strategy combines biomedical evidence with cultural traditions, building trust and creating a lasting impact on oral cancer prevention.

## CONCLUSIONS

Cultural practices such as areca nut chewing, toombak, khat use, reverse smoking, and hot mate drinking remain important yet underrecognized drivers of oral squamous cell carcinoma worldwide. Their persistence reflects deep cultural and socioeconomic roots, demanding prevention strategies that respect tradition while reducing carcinogenic exposure. Dermatologists, with their expertise in mucocutaneous oncology, are uniquely positioned to identify early lesions and counsel patients on risk reduction, particularly in high-prevalence and immigrant communities. Integrating oral mucosal screening into dermatology settings offers an effective, low-cost prevention strategy in high-risk populations.

While this review highlights emerging patterns, the current evidence presented remains limited by several factors. The evidence reviewed here is heterogeneous and varies substantially by exposure. Some associations, such as those involving areca nut and toombak, are supported by strong epidemiologic and mechanistic data; notably, areca nut is formally classified as an IARC Group 1 carcinogen. In contrast, other exposures, including khat, rely on more limited or inconsistent human evidence. Many studies are observational, region-specific, and subject to confounding from co-exposures such as tobacco, alcohol, and socioeconomic disadvantage. There is also considerable variability in how exposure intensity and duration are defined across studies, which makes it challenging to directly compare findings. The mechanisms driving carcinogenesis are not uniform and instead vary by exposure type. For instance, thermal injury is more relevant in the setting of hot beverage consumption and certain chewing practices, whereas nitrosamine-mediated carcinogenesis is more closely associated with smokeless tobacco products such as toombak, and fibrosis-related malignant transformation is most strongly linked to areca nut use. Moving forward, research should focus on prospective study designs, more precise characterization of exposures, and clearer dose-response relationships, in addition to mechanistic studies that better reflect these exposure-specific differences. There is also a need for more community-based intervention research to identify culturally tailored prevention strategies that are both effective and sustainable across different populations. By embedding culturally sensitive education and screening programs within dermatology and public health frameworks, global efforts can reduce OSCC incidence, narrow health disparities, and preserve community identity.
